# Strong magnetic field effect on the nucleation of a highly undercooled Co-Sn melt

**DOI:** 10.1038/s41598-017-05385-y

**Published:** 2017-07-10

**Authors:** Jun Wang, Yixuan He, Jinshan Li, Hongchao Kou, Eric Beaugnon

**Affiliations:** 1 State Key Laboratory of Solidification Processing, Northwestern Polytechnical University, Xi’an Shaanxi, 710072 China; 2 Univ. Grenoble Alps, LNCMI, F-38000 Grenoble, France; 3 CNRS, LNCMI, F-38000 Grenoble, France

## Abstract

High magnetic field is a powerful tool to tune the microstructure and improve the properties of materials. In this report, the nucleation behavior of undercooled Co_76_Sn_24_ near eutectic alloy under strong homogeneous and gradient magnetic fields have been investigated using glass slag fluxing method in a 12 T superconducting magnet. The mean undercooling of the undercooled melt is not altered by homogeneous magnetic field but depressed by gradient magnetic field. The highest temperature during recalescence is strongly altered by magnetic field, where an enhancement effect is observed under gradient magnetic field and an opposite effect in homogeneous magnetic field. The reason is interpreted by discussion about the magnetic field on the thermodynamics of nucleation and also the purifying effect of the glass slag, the magnetic properties and the magnetic force exerted on the undercooled melt.

## Introduction

Non-equilibrium solidification, e.g. rapid quenching, has been successfully applied to produce many metastable solids from the liquid state during the past few decades^1, 2^. One specific method called supercooling method, which can thermodynamically cool the alloy melts (can up to a few hundred K) below its melting point, was found to be a very efficient way to achieve rapid solidification even with very slow cooling rate^3^. The undercooled melt corresponds to a non-equilibrium state of metastable nature as the driving force for crystallization is accumulated due to the Gibbs free energy difference with the increasing undercooling, thus a number of possible solidification paths can occur due to the lower energy of many metastable solids. All these make the investigation of the solidification from undercooled melt interesting and significant.

High magnetic field (HMF) has attracted global attention due to its wide application in materials processing ever since the commercial superconducting magnet became more easily available^4, 5^. A new research area called electromagnetic processing of materials (EPM) becomes a cutting-edge technique and many novel phenomena have been found when the material was processed in magnetic fields, e.g. Levitation^6^, strong magnetized liquid^7, 8^, texturing and orientation^9–13^, change phase transition thermodynamics^14, 15^, controlling the convection^16^. However, up to now, there are only a few works have been done concerning the non-equilibrium solidification of undercooled alloys under magnetic field. Hasegawa *et al*.^17^ studied the undercooling of Cu in 0.5 T magnetic field and found the maximum undercooling was increased when magnetic field was applied and the magnetic field can suppress the irregular and unexpected large decrease of undercooling. By designing a levitation coil in a 10 T magnet, Yasuda *et al*.^18, 19^ found that the convection in the levitated Cu and Cu-1%Ag melt was remarkably reduced but the nucleation temperature was not affected by the field. Zhang *et al*.^20, 21^ found the mean undercooling of Cu increases with increasing magnetic field whereas the mean undercooling of liquid Ge does not show any significant changes by glass fluxing of Cu and Ge in magnetic field. Tsukada *et al*. found Co-rich phase separated from undercooled Co-Cu alloys in high magnetic field^22^. To further uncover the nucleation mechanisms in strong magnetic fields, in the present paper, the effects of strong static magnetic field (includes homogeneous and gradient magnetic field) on the undercooling behavior of Co-Sn near eutectic alloy have been investigated.

## Results

**Figure 1:**
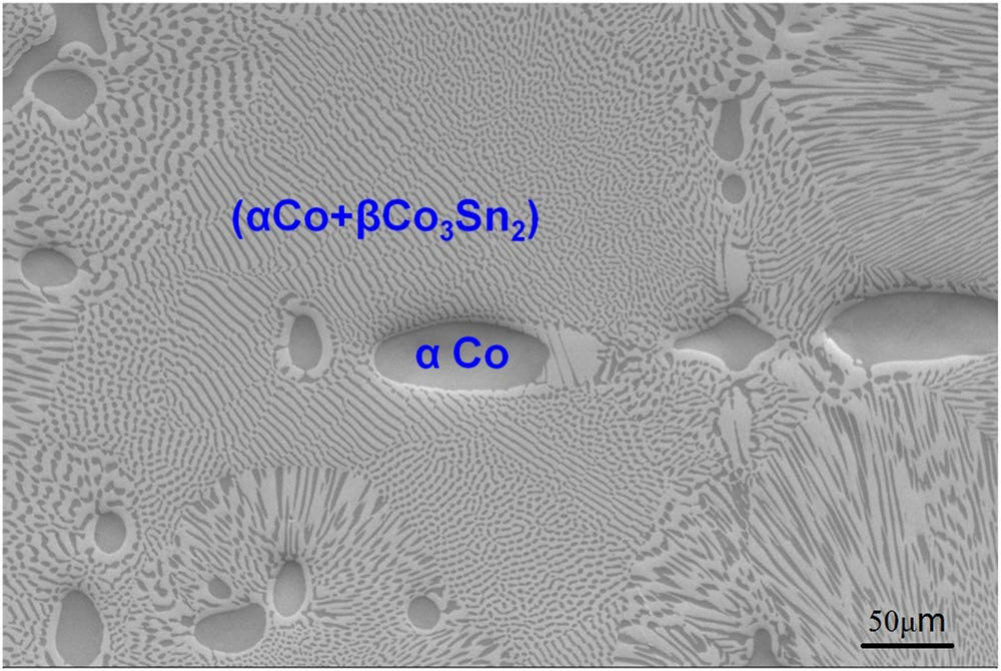
Typical microstructure of Co_76_Sn_24_ near eutectic alloy solidified without magnetic field at low undercooling. The microstructure shows typical hypoeutectic solidification with a small amount of primary α Co phase (dark phase) and lamellar eutectics of α Co (dark phase) + β Co_3_Sn_2_ (white phase).

The microstructure of near eutectic Co_76_Sn_24_ alloy solidified without magnetic field at low undercooling close to its melting point is shown in Fig. 1. The alloy composition, Co_76_Sn_24_ (at. %), is shown to be hypereutectic in Co-Sn binary phase diagram^23^, but determined to be the eutectic point by Liu and Li *et al*.^24^. According to Fig. 1, this composition is very close to the eutectic point and lies in the hypoeutectic range since the microstructure is dominated by regular eutectics (α Co + β Co_3_Sn_2_) and contains a small volume fraction of primary α Co phase. The two phases, α Co and β Co_3_Sn_2_, have quite different magnetic properties, and α Co is found to be ferromagnetic once the temperature is below 1329 K^23^ while β Co_3_Sn_2_ is paramagnetic phase. This makes the phases response quite different when expose to external magnetic fields.

**Figure 2:**
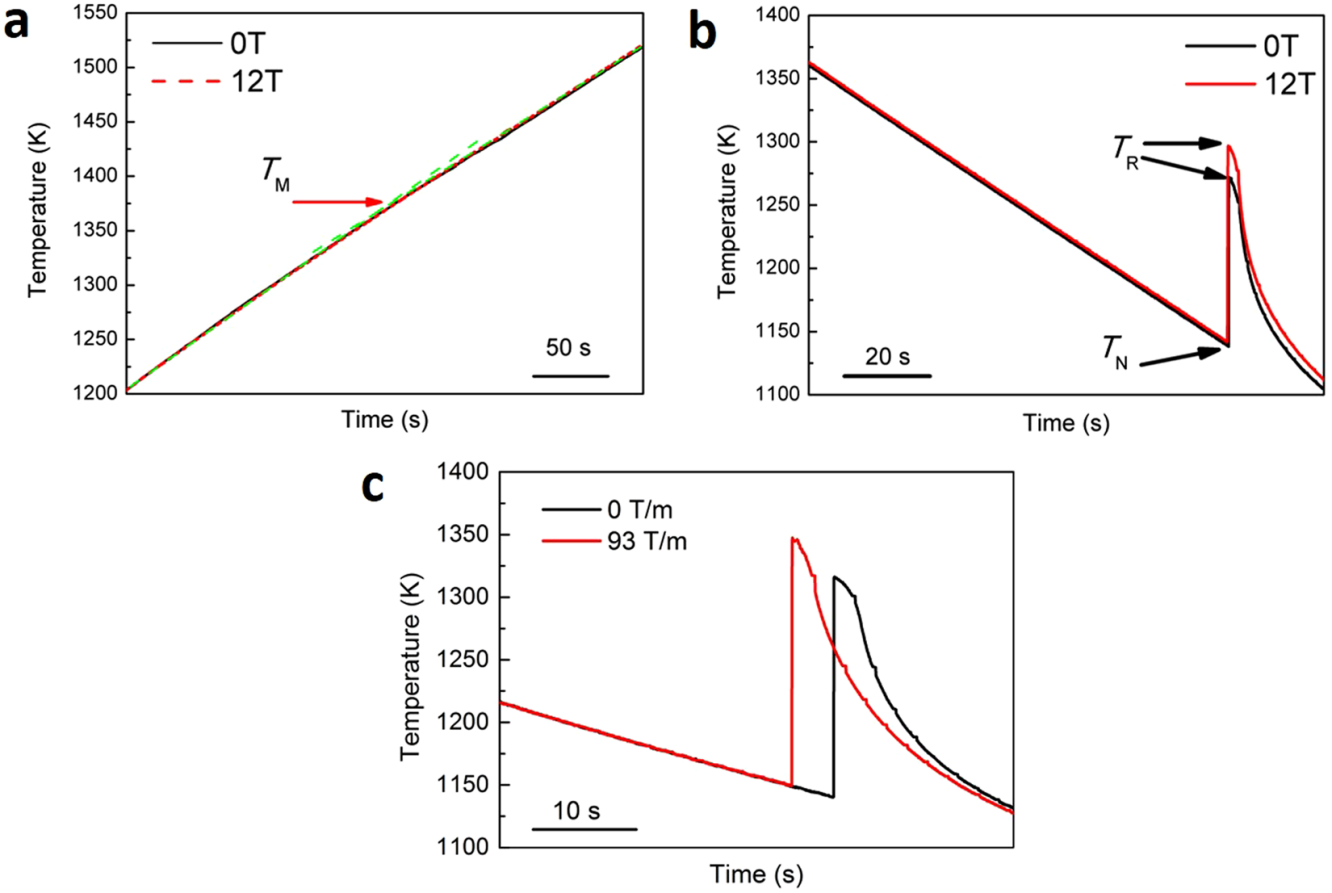
Typical temperature-time curve of Co_76_Sn_24_ near eutectic alloy measured in and without magnetic field. (**a**) The heating curves showing the melting point, the cooling curves showing the recalescence event, (**b**) in homogeneous magnetic field and (**c**) in gradient magnetic field. The melting point is not so evident but can be determined from the slope variations. The application of magnetic field has very limited effect on the melting *T*_M_ during heating and nucleation *T*_N_ during cooling process since the red and black curves are almost overlapped, but we can see difference of the maximum temperature *T*_R_ during recalescence process.

The temperature profiles of Co_76_Sn_24_ near eutectic alloy solidified in and without magnetic field are shown in Fig. 2(a,b) (homogeneous magnetic field) and (c) (gradient magnetic field). During heating process, the temperature profiles measured in and without 12 T magnetic field are almost in an overlapped trace and we cannot find obvious plateau for the melting point. When we look up into the heating curve, two slopes can be determined, and the turning point, 1385 ± 5 K, is assumed to be the melting point. During the cooling process, the temperature traces are almost overlapped before nucleation, shown in Fig. 2(b). The above results indicate that magnetic field has very limited effects on the heating or cooling kinetics of Co_76_Sn_24_ alloy no matter it is in solid or liquid state under homogeneous magnetic field. The recalescence in gradient magnetic field, shown in Fig. 2(c), is different with that in uniform magnetic field where an obvious difference of nucleation temperature is evidenced, and the nucleation temperature is almost the same as that without magnetic field.

**Figure 3:**
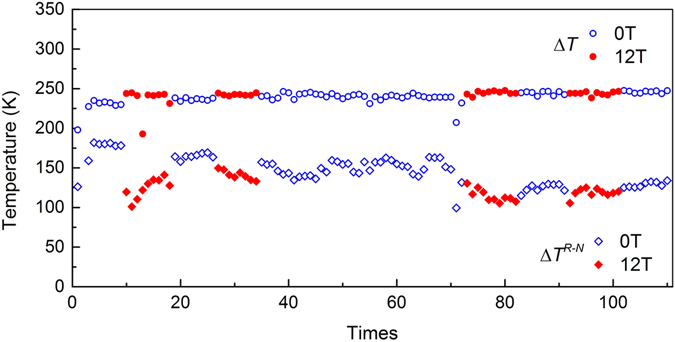
Undercoolings of Co_76_Sn_24_ near eutectic alloy measured in and without 12 T homogeneous magnetic field during cyclic heating process.

Figure 3 shows the undercooling (Δ*T*, defined as the temperature difference between the melting point and nucleation temperature: *T*_M_-*T*_N_) and maximum temperature increase during recalescence (Δ*T* ^*R-N*^, defined as recalescence extent: *T*_R_ *-T*_N_) as a function of cyclic heating times of Co_76_Sn_24_ near eutectic alloy in 0 and 12 T homogeneous magnetic field. It can be seen that the undercooling in and without magnetic field does not show any complicated trend with the increasing cyclic heating times. After vibration at the first few cycles, the undercooling is stable at certain value, and afterwards keeps constant (except for a few abnormal data point below 200 K) regardless of cyclic heating times and magnetic field. The mean undercooling determined is about 245 K.

The recalescence extent, Δ*T* ^*R-N*^, shows different behavior where fluctuation happens when the field intensity changes. When magnetic field is applied (shown in Fig. 3), Δ*T* ^*R-N*^ will decrease to a lower value. The mean value of Δ*T* ^*R-N*^ for the first 10^th^ cycles in zero magnetic field is 177 K while decreases to 134 K for the following 10 cycles when 12 T magnetic field is applied. With the increasing heating cycles, the difference becomes smaller, as shown for the last variations of the field intensity, from 128 K at 12 T magnetic field to 119 K without magnetic field. Thus, it can be concluded that even the magnetic field has very limited effect on the mean undercooling of the undercooled melt, the recalescence extent which is a signal showing the maximum heat release of the solidification can be depressed by the magnetic field.

**Figure 4:**
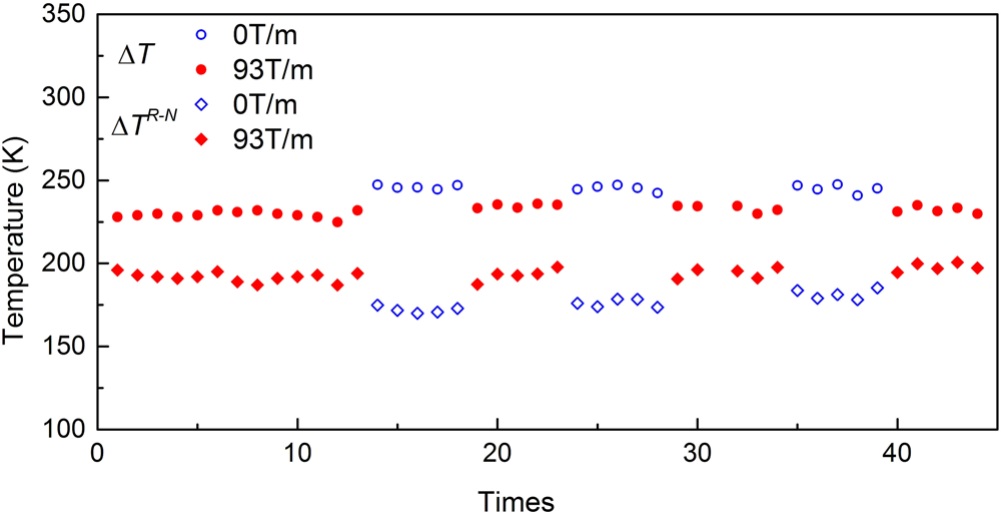
Undercoolings of Co_76_Sn_24_ near eutectic alloy measured in alternately magnetic gradient fields.

Figure 4 illustrates the undercooling as a function of cyclic heating times of Co_76_Sn_24_ near eutectic alloy in 0 and 93 T/m gradient magnetic field. Different from the trend shown in Fig. 3, the undercooling decreases when the external gradient field is applied. The mean undercooling calculated in 0 and 93 T/m gradient magnetic field are 246 K and 232 K, respectively. That is to say, the application of gradient magnetic field can depress the mean Δ*T*.

The recalescence extent, Δ*T* ^*R-N*^, shows opposite trend behavior compared to that in homogeneous magnetic field where an increasing trend is evidenced from 177 K to 194 K when a 93 T/m gradient magnetic field is applied.

## Discussions

**Figure 5:**
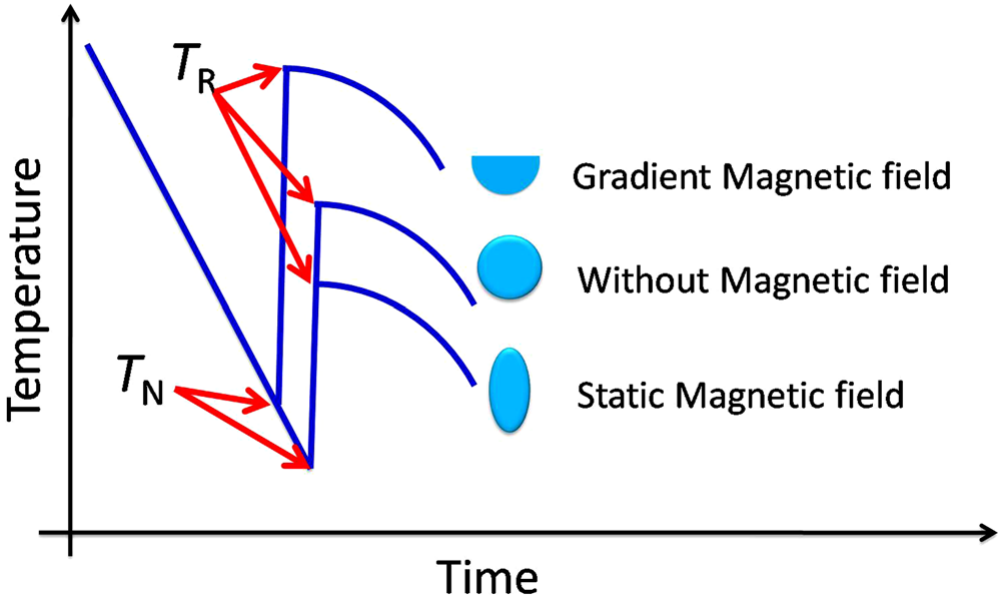
Schematic figure showing the recalescence and sample shape solidified under different conditions.

Figure 5 is the schematic figure showing the temperature profile of recalescence behavior and also the sample shape solidified under different magnetic field conditions. The sample solidified in gradient magnetic field has a lower undercooling but highest recalescence extent with a semi-spherical bottom and flat top surface sample. The samples solidified without (spherical shape) and with homogeneous 12 T magnetic field (ellipsoidal like shape, with with ellipticity (c/a) close to 0.7) have the same undercooling, but the latter has a much lower recalescence extent. Next we will discuss the factors that can cause these differences.

**Figure 6:**
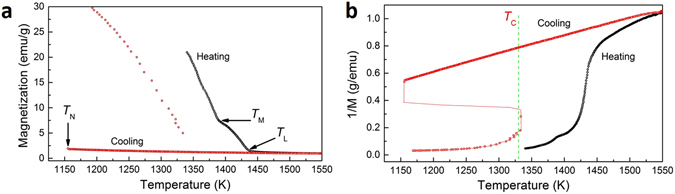
(**a**) Magnetization as a function of temperature curves and (**b**) Inverse Magnetization as a function of temperature curves of Co_76_Sn_24_ near eutectic alloy measured in 93 T/m gradient magnetic field and the field intensity is 6.23 T at the sample position.

Figure 6(a) is the magnetization as a function of temperature during heating and cooling process for Co_76_Sn_24_ alloy in 93 T/m gradient magnetic field (the field intensity at the sample position is 6.23 T). During heating process, the magnetization will decrease with the increasing temperature. *T*_M_ detected by magnetization curve is about 1385 K which is consistent with the melting point shown in Fig. 2(a). Above *T*_L_ (1437 K), the alloy turns into fully liquid state and during the cooling process, the magnetization curves are overlapped for the data measured during heating. When the melt enters into the undercooled state, we do not see any kink point and the curve still follows the same trend and the discrepancy between the heating and cooling process becomes larger before the nucleation starts at *T*_N_ = 1152 K: the magnetization of the undercooled liquid is much smaller than its solid state at the same temperature. The trend can be made clearer when we plot the inverse magnetization curve as a function of the temperature (shown in Fig. 6(b)). The melt will be in paramagnetic state in case of the 1/M-T curve is linear according to the Curie-Weiss law. During heating process, the linear state initiates when the temperature is far above the melting point while during the cooling process, the whole curve is in the same slope before the nucleation happens.

After solidification, two phases, ferromagnetic α Co and paramagnetic β Co_3_Sn_2_ phase are formed in Co_76_Sn_24_ near eutectic alloy. Compared to α Co, the magnetic Gibbs free energy of β Co_3_Sn_2_ can be neglected due to its low magnetic susceptibility. In the present experiment, when Co_76_Sn_24_ alloy is solidified at undercoolings shown in Fig. 3, the solid α Co phase will be in ferromagnetic state due to the temperature is blow the Curie point (even there may be a few seconds above the Curie point during recalescence). The Gibbs free energy of undercooled Co_76_Sn_24_ alloy melt in magnetic field can be expressed as follows:

1 $${\rm{\Delta }}G={\rm{\Delta }}{G}_{V}+{\rm{\Delta }}{G}_{M}$$

2 $${\rm{\Delta }}{G}_{V}=\frac{{\rm{\Delta }}{H}_{f}{\rm{\Delta }}T}{{T}_{m}}$$

3 $${\rm{\Delta }}{G}_{M}=-{\mu }_{0}{\int }_{0}^{H}{({M}^{S}-{M}^{L})|}_{T={T}_{N}}\,d{H}_{ex}$$

where ∆*G*_*V*_, ∆*G*_*M*_ are chemical Gibbs free energy and magnetic Gibbs free energy, ∆*H*_*f*_, *μ*_0_, *M* ^*S*^, *M* ^*L*^, *H*_*ex*_ are melting enthalpy, permeability of vacuum, magnetization of the solid and liquid and the external magnetic field, respectively.

**Figure 7:**
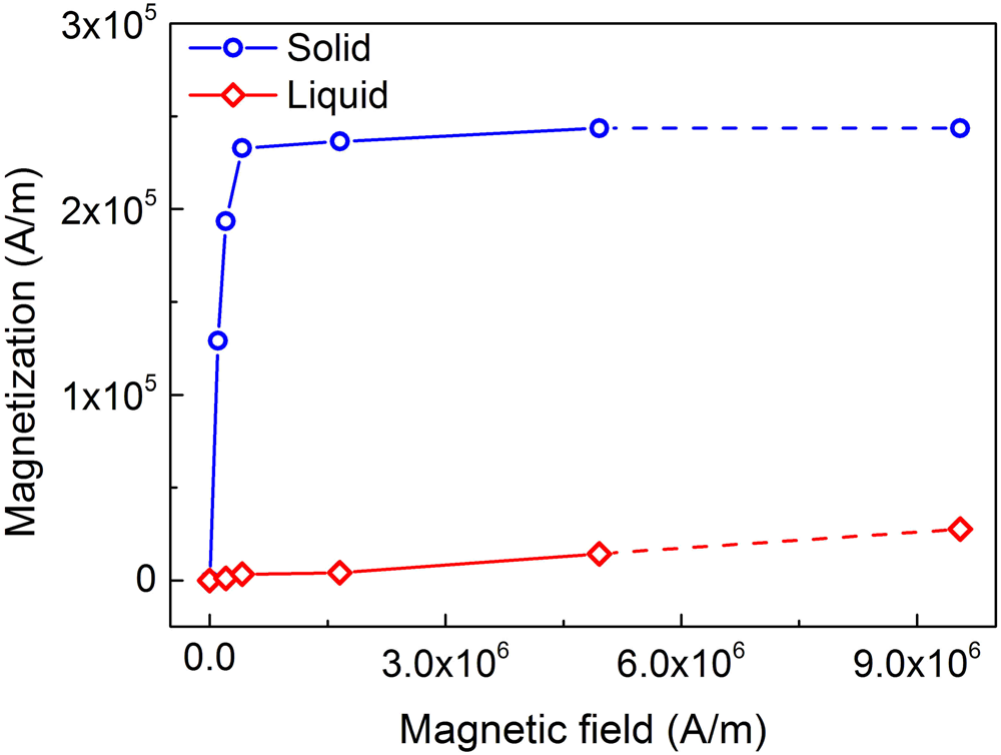
The magnetization of Co_76_Sn_24_ during heating (in solid state) and cooling (in uncercooled liquid state) at 1152 ± 2 K under different external fields.

By using the parameters, *T*_*M*_ (1385 K), Δ*H* (13.027 kJ/mol), Δ*T* (245 K), and magnetization shown in Fig. 7, the calculated magnetic and chemical Gibbs free energies are ∆*G*_*M*_ = 28 J/mol and ∆*G*_*V*_ = 2304 J/mol, respectively. The application of magnetic field just add about 1% of the total energy of the system, so that its effect on the thermodynamics of nucleation is very weak.

Thus from thermodynamic point, the magnetic field, either in homogeneous or gradient magnetic field, has quite limited effect on the undercooling. According to Fig. 3, it is reasonable to understand the small undercooling variations when homogeneous magnetic field is applied. However, the strong depression effect shown in Fig. 4 still needs to turn to other effect, e.g. magnetic force exerted on the sample, which will be discussed later.

According to the nucleation theory, the ordering structure in the melt was the precursor of crystals. Even though it is stressed that metallic melts possess their own characteristic ordering structure that is usually independent of the corresponding solid phases, the X-ray diffraction results^25^ showed that the near eutectic Co-Sn alloy holds quite stable β Co_3_Sn_2_ and α Co clusters in the overheated melts which is consistent with its solid structure. Thus, in the liquid Co-Sn alloy, ferromagnetism could arise when ferromagnetic long range ordering structure is formed. However, according to Fig. 6, it can be seen that during the whole liquid state of Co-Sn alloy melt, even it enters into the undercooled range, the linear 1/M-T curve shows that the melt is always in the paramagnetic state before nucleation happens. So the maximum nucleation temperature was not visibly affected by the external field since the undercooling did not surpass the Curie point at which the magnetic contribution could change the nucleation temperature dramatically^26^. However, it is not easy to undercool the alloy to such extent of Co-Sn alloy below its liquid Curie temperature by present technique.

**Figure 8:**
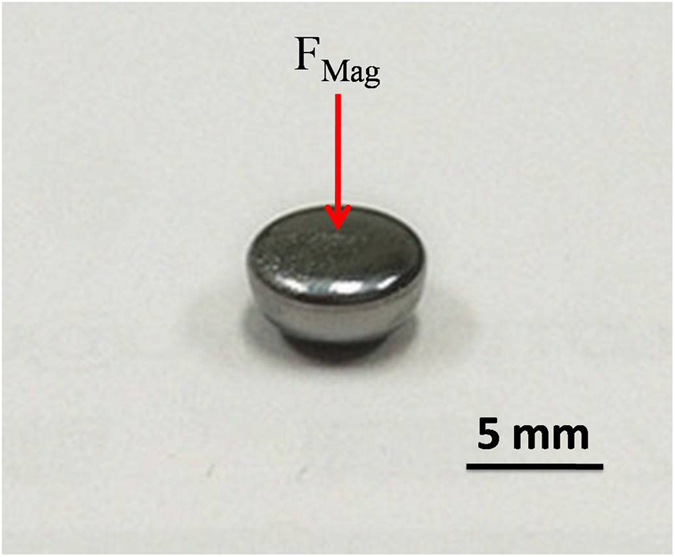
Image of the sample solidified in strong gradient magnetic field of Co_76_Sn_24_ alloy.

In homogeneous magnetic field, the undercooling keeps unchanged while in gradient magnetic field, the mean undercooling decreases 14 K. According to the magnetization measurement (shown in Fig. 6(a)), Co_76_Sn_24_ undercooled melt is still in paramagnetic state, however, the undercooled melt bears a magnetic force in strong gradient magnetic field which can change the shape of the sample to a compressed pie shape (shown in Fig. 8). Thermodynamically, magnetic field has very limited effect on the variation of the undercooling of Co-Sn near eutectic alloy, no matter in homogeneous or gradient magnetic field. Here, for Co_76_Sn_24_ alloy, the paramagnetic state of the sample let the field exert a compressing force along the field direction (about 17 times of its weight, which is directly measured by the Faraday balance installed on top of the magnet) while the diamagnetic fluxing glass is experiencing a very weak upward force at the same gradient field, which means the purifying effect of the glass slag on the undercooled melt is weakened. This effect can make the undercooling smaller.

The sample shape variation also can destroy the equilibrium between the sample and glass slag, e.g. the wetting angle is altered^27^ and the surface tension between the melt and the glass slag is enlarged when the shape changed from spherical to a pie like shape, which can decrease the mean undercooling. In a word, the sample processed in gradient magnetic field can undergoes strong magnetic force that can lead to a lower undercooling.

The recalescence extent is interpreted by two factors: the total energy released during recalescence and the heat release speed. The total energy is always treated as the enthalpy of fusion for the common solidification process. In this case the application of magnetic field can thus change the energy of the system, but have quite limited effect as discussed above.

During the recalescence process from deep undercooled melt, the latent heat is released in a few tens millisecond, which can be treated as adiabatic process due to the release of latent heat is much larger than the radiation of the heat by the melt to the environment. Hence the latent heat released depending on the volume fraction of the solidified phase during rapid solidification, the solidification fraction during recalescence can be used to estimate the total heat released. When the undercooling is above the hypercooling limit, all the latent heat will be absorbed by the liquid phase and there is no heat left for releasing to the environment. The hypercooling limit of Co_76_Sn_24_ alloy calculated by Δ*T*_hyper_ = Δ*H*/*C*_P_ is about 386 K which is higher than the undercooling we obtained in the present study (Δ*T* = 245 K). That means not all the liquid will solidify during the recalescence.

**Figure 9:**
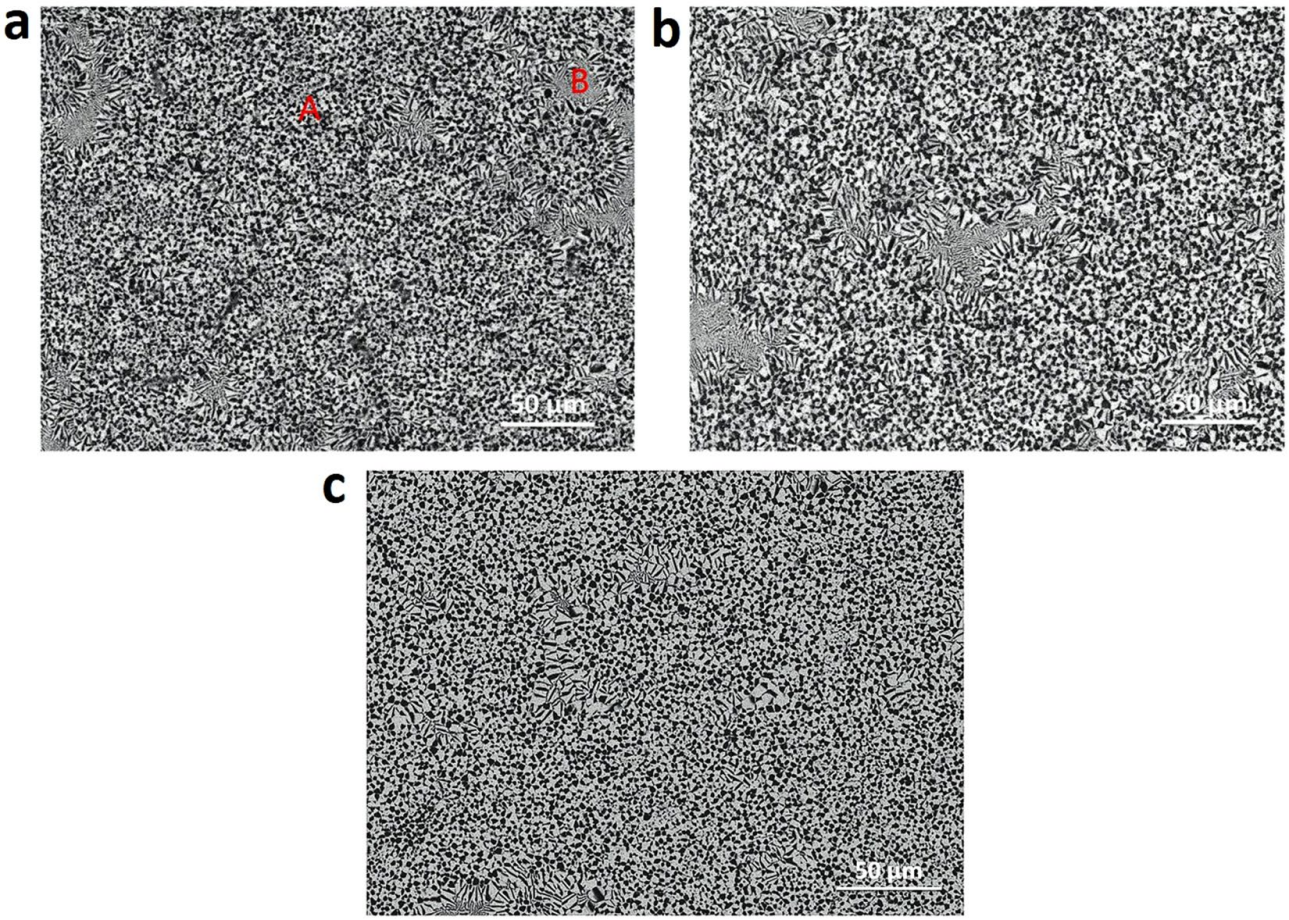
Microstructure of Co_76_Sn_24_ alloy solidified in homogeneous magnetic field (**a**) ΔT = 236 K, B = 0 T, (**b**) ΔT = 240 K, B = 12 T, and (**c**) in 93 T/m gradient field at the undercooling of 232 K.

Figure 9 presents the microstructure of Co_76_Sn_24_ alloy solidified in different magnetic fields at the undercoolings about 232 ± 4 K. Two different morphologies can be observed, anomalous eutectics marked as “A” and lamellar eutectics marked as “B” in Fig. 9(a). The volume fraction of anomalous eutectics can be treated formed during recalescence after nucleation and the regular lamellar eutectics are formed after recalescence when the temperature of the melt is much higher. By calculation, the volume fraction of anomalous eutectics are 95.8%, 96.4% and 99.5% for the alloys solidified without magnetic field (shown in Fig. 9(a)), in homogeneous magnetic field (shown in Fig. 9(b)) and gradient magnetic field (shown in Fig. 9(c)), respectively. Thus during recalescence the volume fraction of the solidification in gradient magnetic field is the highest, and very close in homogeneous magnetic field and without magnetic field. So the total latent heat released takes this order, and *T*_R_ is higher when the released heat is larger. This can be explain why the sample solidified in gradient magnetic has the highest *T*_R_ (shown in Fig. 5). However, still we cannot explain why Δ*T* ^*R-N*^ of the sample solidified in homogeneous magnetic field (Δ*T* ^*R-N*^ = 128 K) is lower than that without magnetic field (Δ*T* ^*R-N*^ = 119 K). One possible explanation is due to the existence of defects (such as dislocation, vacancy) and stress formed in the solid skeleton upon rapid solidification that much energy is saved in the as-formed solid skeleton upon recalescence, which will reduce the latent heat release and consequently, decrease *T*_R_^28^. The alloy solidified in high magnetic field have more defects, which will induce more this kind of energy and lead to a much lower *T*_R_.

## Conclusions

1. The Co_76_Sn_24_ near eutectic alloy can be highly undercooled with mean undercooling about 246 K and 232 K in strong homogeneous and gradient magnetic field.
2. The magnetization of liquid Co_76_Sn_24_ near eutectic alloy shows a paramagnetic behavior in a wide temperature range from superheated to deep supercooled state.
3. Compared to chemical Gibbs free energy, the magnetic field induced energy is so small that its effect on the thermodynamics of nucleation is very weak, which can explain why the mean undercooling are not altered by the high homogeneous magnetic field. The gradient magnetic field will reduce the mean undercooling which is attributed to the magnetic force exerted on the undercooled liquid which can break the purifying and nucleation condition.
4. The gradient magnetic field can increase the maximum temperature during recalescence, which is attributed to the promoted solidified volume fraction during recalescence. The homogeneous magnetic field can decrease the maximum temperature during recalescence, which is thought to relate with the magnetic field induced defects that consumes the energy of the system.

## Methods

### Sample preparation

Co_76_Sn_24_ (at. %) near eutectic alloy was prepared by cold crucible levitation melting method by mixing high purity elements (99.99 wt. %). The obtained ingot was then machined into small pieces with average mass about 1 g to be used as the sample candidate. A sample together with B_2_O_3_ glass slag was inserted into a high purity quartz tube crucible. The whole system was put in a self-designed furnace which was designed in a 12 T superconducting magnet. A two-color pyrometer installed on top of the magnet with measuring frequency of 50 Hz was used to *in-situ* measure the temperature of the sample and an S-type thermocouple installed below the bottom of the quartz tube was used to control the heating and cooling speed of the furnace. A detailed description of the apparatus is shown in Ref. 29.

### Undercooling and measurement for the magnetic force

The undercooling experiments are conducted at homogeneous magnetic field where the field intensity is the maximum and there is no field gradient in the magnet bore and gradient magnetic where the sample is placed above the maximum field of the magnet, at a position where the radial magnetic forces will center it on the axis on the magnet. The magnetization and magnetic force can be *in-situ* measured in gradient magnetic. The vertical magnetic field gradient exerts a downward vertical force that is monitored by an electronic balance placed above the magnet and below which the quartz crucible is suspended. The sample magnetization and magnetic force is then continuously monitored from this force measurement during the melting, overheating and solidification process.
